# An Evaluation of Naloxone Use for Opioid Overdoses in West Virginia: A Literature Review

**DOI:** 10.3844/amjsp.2015.9.13

**Published:** 2015-07-09

**Authors:** Annahita Beheshti, Landyn Lucas, Tanika Dunz, Maryna Haydash, Hope Chiodi, Breanna Edmiston, Chad Ford, Natalie Bohn, John Hunter Stein, Anthony Berrett, Brittney Sobota, Joseph Horzempa

**Affiliations:** 1 Department of Graduate Health Sciences, West Liberty University, West Liberty, WV, USA; 2 Department of Natural Sciences and Mathematics, West Liberty University, West Liberty, WV, USA

**Keywords:** Naloxone, West Virginia, Opiate, Opioid, Heroin, Overdose, Oxycodone

## Abstract

Naloxone is a common treatment option for the reversal of an opioid overdose. The law regulating naloxone varies from state to state and therefore so does the drug’s availability. The state of Rhode Island has legalized naloxone for over-the-counter use while a number of states allow prescriptions to opioid abusers and family members. The medical community as a whole appears to be divided on the issue of availability to lay persons. This is largely due to a lack of information on this relatively new subject, as well as discrepancies within the existing naloxone research in regard to the use of naloxone among the general public. This literature review provides an objective examination of the pros and cons of increasing the availability of naloxone to the public, with an emphasis on the state of West Virginia (WV). Due to WV’s high rates of opioid abuse and overdose, the implications of increasing public accessibility to naloxone could be invaluable in the effort to reduce opioid related deaths. To this end, a WV law that has recently gone into effect allows emergency personnel and family members of opioid addicts to carry naloxone. Ongoing research investigations will determine the impact of this law in regard to the overall wellbeing of the residents of WV and the implications on future laws regulating naloxone use.

## Introduction

Drug overdose is the leading cause of accidental death in the United States (US) and is a global public health issue (Wermeling, 2013). Overdose is defined as the accidental or intentional administration of a drug at a quantity substantially greater than normally used or recommended resulting in serious harmful symptoms or death. The largest proportion of drug overdose is due to opioid misuse (CDC, 2014). While the factors contributing to opioid overdose vary greatly among regions of the US, people in rural communities are more likely to overdose on opioids than those near cities (Levi *et al*., 2013). This geographical disparity indicates that there may be factors associated with a pastoral lifestyle that contribute to overuse of drugs. For instance, West Virginia (WV), a state in which over 50% of the population lives in rural areas, has the highest per capita overdose rate of any state in the nation (US Attorney’s Office, 2011). The rate of misuse of opioids in WV has surged recently with a greater than 6-fold increase in overdoses since 1999 (Drug Abuse, 2013). Prescription drugs attribute to nine of every ten overdoses in WV, which includes the overuse of several opioids such as hydrocodone, morphine and oxycodone (US Attorney’s Office, 2011). This misuse of prescription medication is the top criminal matter in many parts of WV (US Attorney’s Office, 2011) indicating that in addition to affecting human health, drug abuse in WV has deleterious sociological implications.

Naloxone is a well-known antidote for opioid overdose with decades of clinical evidence as an injection-based pharmaceutical. Although the mechanism of action is not fully understood, evidence suggests that naloxone may act as a μ-opioid receptor competitive antagonist (Wermeling, 2013). Because of the apparent benefit of naloxone in preventing accidental death from opioid overdose, US legislatures, including those in WV, have proposed to provide this drug as an Over-The-Counter (OTC) medication (Wermeling, 2013). However, there is little evidence supporting the effectiveness of naloxone in deterring illegal use of opioids. Moreover, many health care professionals do not recommend legalizing naloxone for OTC use. Opponents to naloxone legalization contend that the implementation of overdose prevention programs focusing on education and awareness are safer alternatives that reduce opioid use (Wheeler, 2012).

## Main Text

The purpose of this article is to provide a comprehensive review of the literature, detailing the positive and negative impacts of legalizing OTC naloxone in the state of WV. Current literature and policies relating to naloxone use were identified by searching Pubmed Central, EBSCO, Google scholar, government websites such as NIH.gov, CDC.gov and wvdhhr.org. Keywords used in locating articles included: drug abuse, drug overdose, opioids, Narcan, naloxone, Ohio Valley, West Virginia, Pennsylvania and Ohio. Articles were identified between the dates of 09/29/14 and the date of submission.

### West Virginia Socioeconomic Factors Contribute to Overdose Susceptibility

Behaviors leading to opioid abuse and misuse, which may culminate as an overdose, vary greatly among regions of the US. Naloxone is a well-known antidote that has been used for decades as an injection-based pharmaceutical. In treating narcotic drug overdose, naloxone reverses the systemic effects from narcotics. Opioid drug abuse is an issue that affects the life of citizens of WV and opioid abuse is increasing at an alarming rate. WV has the highest per capita overdose rate in the nation (US Attorney’s Office, 2011). Nine of every ten overdoses involve prescription drug abuse, of which includes the overuse of several opioids such as oxycodone (CDC, 2014). Contrary to the general perception that drug use is more common in large cities, people in rural communities are actually more likely to overdose on opioids than people in urban communities (Drug Abuse, 2013). As a substantially high number of WV citizens are on worker’s compensation programs, the overuse of painkillers to treat the chronic pain of these individuals is becoming more frequent (Drug Abuse, 2013). The overwhelmingly rural population coupled with high volume of individuals on worker’s compensation programs results in the population of WV being especially vulnerable to the risk of opioid misuse and overdose.

### Opioid Abuse in West Virginia

Opioid drug abuse has become an increasing problem in WV (Drug Abuse, 2013). In 2010, the nationwide age-adjusted drug poisoning death rate showed WV to have the highest at 28.9 per 100,000 people, which has quadrupled since 1999 (Drug Abuse, 2013). Unintentional overdoses surpassed motor vehicle accident deaths for the first time in WV in 2008 and even more so in 2010 (WVU-ICRC, 2013). Moreover, 137.6 opioid prescriptions were written per 100 people in WV in 2013, which is the third highest rate of any state with only Alabama and Tennessee having a higher rate (CDC, 2014). The most commonly abused opioids are the prescription drugs, Vicodin® (acetaminophen and hydrocodone) and OxyContin®(oxycodone) (NIH, 2014). To assist WV in resolving this issue of opioid overuse, the CDC has awarded over a million dollars to this state to be paid over the next three years to improve the drug-monitoring program for prescription drugs (CDC, 2014).

### Naloxone Background

Naloxone, a pure opioid antagonist, is often used in conjunction with emergency medical treatment for the reversal of life-threatening effects of opiate overdoses. While the mechanism of action of naloxone is not fully understood, evidence suggests that this drug antagonizes the opioid effects through competitive inhibition for μ, κ and σ receptor sites in cells of the central nervous system (PubChem Compound Database, 2014).

Naloxone is typically administered intravenously, intramuscularly, or subcutaneously (Clarke and Dargan, 2002). The activity of naloxone may cause a complete reversal of narcotic effects. This abrupt change could result in acute withdrawal syndrome which involves tremulousness, nausea, vomiting and more severe symptoms such as tachycardia and cardiac arrest (DailyMed, 2013). Therefore, naloxone should be carefully administered to persons, including newborns, who are suspected or acknowledged to be physically reliant on opioids. A patient who has responded well to naloxone should be continuously monitored, since the duration of action of some opioids may be greater than that of naloxone (DailyMed, 2013). Since the half-life of naloxone in adults ranges from 30 to 81 minutes (DailyMed, 2013), proper administration of this antidote often involves repeated injection. The short duration of naloxone coupled with the fact that some narcotics may persist beyond the therapeutic capacity of this antidote could necessitate re-administration. An initial dose of 0.4 mg to 2 mg of naloxone may be administered and if the desired improvement in the patient is not obtained, re-administration of the drug may be necessary, given in 2 to 3 min intervals (DailyMed, 2013).

Emergency medical staff, as well as law enforcement personnel, typically carry intravenous naloxone as this is the quickest route for administration in emergency situations (ASHSP, 2014). In addition, family members of opioid addicts are now permitted to carry naloxone in WV. Naloxone is stored at room temperature (15–25°C) and housed in a protective outer case (Kaleo, 2014). This packaging renders naloxone impact-resistant making this drug safely transportable by emergency medical staff. Individuals addicted to opioids can be prescribed naloxone and may auto-inject this antidote (ASHSP, 2014). Here, verbal instructions provide information on self-inoculation of the drug upon opioid overdose (ASHSP, 2014). Intranasal instillation is a non-injection based option, which eliminates the risk of accidental contaminated needle sticks to health care individuals (Wermeling, 2013). Administration of a naloxone nasal spray produces a systemically-active blood concentration without damaging the upper respiratory system (Wermeling, 2013).

### Current Guidelines

Recent legislation across the US has addressed the distribution and administration of naloxone. Twenty-four states have generated laws allowing physicians to prescribe naloxone to opioid abusers to reverse overdose (Campo-Flores and Elinson, 2014). Moreover, seventeen states have also passed “Good Samaritan” laws that allow physicians to prescribe naloxone to the family members of abusers (Campo-Flores and Elinson, 2014). Because of the effectiveness in reversing opiate overdose, many states are campaigning to make naloxone more easily accessible. Rhode Island recently (August 2014) passed a bill that legalized the OTC naloxone, increasing public accessibility. Presently in WV, emergency personnel (police officers, EMS, firefighters) and family members of opioid addicts trained in the use of naloxone are permitted to carry this remedy.

### Risks and Benefits of Naloxone Use

Naloxone has been used to reverse over 10,000 opioid-related overdoses since initial use of this drug in the USA (Wheeler, 2012). However, because legalization of OTC naloxone in states such as Rhode Island is in its infancy, statistics describing the impact of usage by the general public are not yet available. Ohio, a neighboring state of WV, is one of several states that allows third party prescribing of naloxone and immunity from criminal liability for the distributor (Davis, 2014). Advocates of naloxone push for legislation legalizing OTC use as these individuals argue that visiting with a physician hinders the potential of naloxone success (Massatti, 2013). Furthermore, advocates of OTC naloxone insist this antidote is safe and can be administered with minimal side effects other than those associated with opiate withdrawal (Massatti, 2013). However, the availability of an antidote may encourage drug use by providing a sense of security in users. In Ohio, rates of naloxone administration have increased 38% from 2003 to 2012 (Massatti, 2013). Diagnoses of opioid addiction and abuse increased almost 330% from 2001–2012 (Massatti *et al*., 2014). This surge in opiate addiction correlates with the increase in naloxone usage associating these two phenomena.

Fifty-nine percent of people using naloxone in Ohio are between the ages of 25–54 (Massatti, 2013; Christiadi *et al*., 2014). Moreover, Caucasians and African-Americans had the highest rates of naloxone use in Ohio (Massatti, 2013). These demographics are the most prominent in WV as well, indicating that abundant naloxone use could potentially occur in WV if similar policies existed (US Census, 2014). Additionally, the rate of naloxone use has increased for every age group from 2003–2012 in Ohio suggesting that relaxed laws governing the use of naloxone may lead to increased use (Massatti, 2013).

Even considering the potential benefits of OTC naloxone reducing the number of opioid-associated deaths, many argue that this remedy would be used as a safety net, thus leading to heavier drug use and dependence among abusers (Seal *et al*., 2003). When 82 heroin users were surveyed, only 35% reported that they would be likely to use this drug if they had access to naloxone (Seal *et al*., 2003). In this same study, 87% reported that they would be willing to participate in an overdose management training program and 84% would carry naloxone if they received training (Seal *et al*., 2003). The Center for Disease Control and Prevention (CDC) Morbidity and Mortality Weekly Review (MMWR) also advocates education of opioid overdoses and naloxone use to drug abusers because doing so can decrease the rate of death among opioid overdose victims (Wheeler, 2012). Since naloxone has been reportedly used to save over 10,171 lives in the US (Wheeler, 2012), substantially fewer overdose related fatalities may occur if naloxone were made available OTC.

## Discussion

Despite the understanding that naloxone has saved many lives, the general medical community is divided on whether this drug should be widely available to everyone (Khoury, 2014). Opponents to legalization contend that OTC naloxone will promote the use of opioids. In addition, if naloxone is more readily available, those opposed to OTC legalization argue that users will take greater risks, such as higher doses and increased frequency of use. There is concern that untrained persons lack the knowledge to recognize an overdose, which leads to a misuse of resources (WVU-ICRC, 2013). Furthermore, once the effects of naloxone wear off, there is a possibility for respiratory depression to occur which is a condition in which breathing is reduced to below 12 breaths per minute (WVU-ICRC, 2013). Finally, bystanders may be less willing to call for emergency assistance if they think they have the cure for the overdose (WVU-ICRC, 2013).

Evidence suggests that people who misuse opiates will make efforts to ensure that they use these drugs in areas where naloxone is administered by a healthcare professional. Although there is evidence suggesting that naloxone prevents many overdose-induced deaths, the number of overdoses and uses of naloxone have risen drastically since the introduction of naloxone (Massatti, 2013). A study in Ohio among Emergency Medical Service (EMS) personnel found that once opiate users were aware the EMS staff carried naloxone, drug abusers more frequently put themselves in situations where access to responders carrying this antidote was guaranteed (Massatti, 2013). Some documented instances included abusers crossing state lines to do their drugs because they knew naloxone would be available if needed (Massatti, 2013). Many overdoses occur from intentional drug abusers and Good Samaritan laws (laws protecting those who provide reasonable assistance to the incapacitated) do not exist to protect the abusers and associate bystanders from legal action for use or possession of opiates. Therefore, when EMS arrives to the scene, oftentimes the overdose victim has abandoned by fellow users as these individuals flee to avoid punitive action (Massatti, 2013). If naloxone were to be available over the counter, sufficient use of 911 emergency could diminish and the incidence of overdose will likely continue to increase (Massatti, 2013).

Education and community-based programs have been effective at reducing opioid overdoses. For instance, over 10,000 overdose reversals can be attributed to community-based overdose prevention programs since 1996 (Wheeler, 2012). Moreover, a recent pilot study indicated that participants who just received overdose education versus those receiving overdose education in addition to naloxone rescue kits did not differ in opioid use, overdose rate, or response to a witnessed overdose indicating the potential effectiveness of education (Dwyer *et al*., 2015). Fewer than three of these drug overdose prevention programs distribute naloxone in WV, yet 188 are known across the US (Wheeler, 2012). Despite WV having the highest rate of overdose deaths in the country (as of 2008), this state only has 1.6% of the programs aimed at preventing overdose related deaths (Wheeler, 2012). Clearly, this indicates a flaw in the efforts to prevent these accidental overdoses. For this reason, even if naloxone is not made more accessible to drug abusers, overdose-associated deaths would be reduced if more overdose prevention programs were established in WV. Regardless of the potential negatives associated with naloxone use by the general public, this antidote has been shown to be effective in preventing opioid-associated deaths when in the hands of trained individuals.

Because OTC naloxone is a novel concept, the amount of controlled research studies to gain an indication of future implications is limited. However, several issues have been brought to the attention of the FDA and WV state legislature. The first being that an abuser wanting to self-administer naloxone may not accurately understand the label information and the instructions for use (Leonard-Segal 2012). If the label and instructions cannot be interpreted, potential misuse may arise. The second is the rapid onset of serious withdrawal symptoms. Here, the intensity of withdrawal can be serious enough to require medical attention and leave the user incapacitated. Therefore, if the person who has overdosed is alone, they may be unable to contact necessary help. Lastly, if legislation were to pass the allowance of OTC naloxone, an increase in drug abuse could occur. Despite the potential for negative consequences, naloxone has reversed over ten thousand opioid overdoses. While many of these consequences could be remedied by a publicly offered educational course prior to purchasing the drug (Maryland Department of Health and Mental Hygiene, 2014), more research in states that allow naloxone to be purchased OTC is necessary. Analyzing the impact of OTC naloxone will allow legislators in WV to base law on evidence regarding the safety and impact of this opioid overdose remedy.
